# A randomized, double-blind, placebo controlled safety, tolerability, and pharmacokinetic dose escalation study of a gentamicin vancomycin gel in patients undergoing colorectal surgery

**DOI:** 10.1186/s13741-016-0043-2

**Published:** 2016-06-16

**Authors:** Elliott Bennett-Guerrero, Harold S. Minkowitz, Alvaro M. Segura-Vasi, Jorge E. Marcet, Jennifer A. White, G Ralph Corey, Kent S. Allenby

**Affiliations:** Duke Clinical Research Institute, Duke University, Durham, NC USA; Memorial Hermann Memorial City Medical Center, Houston, TX USA; Shoals Clinical Research, Florence, AL USA; Tampa General Hospital, Tampa, FL USA; Dr. Reddy’s Laboratories, Inc., Princeton, NJ USA

**Keywords:** Surgical site infection, Colorectal, Topical antibiotic, Gentamicin, Vancomycin

## Abstract

**Background:**

Despite numerous interventions promulgated by the Surgical Care Improve Project (SCIP) and other organizations, surgical site infection (SSI) continues to be a significant medical problem. DFA-02 is a novel bioresorbable modified-release gel consisting of both gentamicin (16.8 mg/mL) and vancomycin (18.8 mg/mL) to be applied during surgical incision closure for the prevention of SSIs. The following double-blind phase 2a trial was designed to test the safety and tolerability of DFA-02.

**Methods:**

At six US sites, the study planned to randomize 40 subjects undergoing colorectal surgery (30 with DFA-02, and eight with placebo gel) in four ascending dose cohorts (10-, 20-, 30-, and 40-mL study drug per wound). Safety was ascertained and serum pharmacokinetics (PK) was determined.

**Results:**

Study enrollment was discontinued after the first three dose cohorts (10, 20, and 30 mL) as even very large incisions could not accommodate more than 20 mL of gel, leaving no scientific justification for the 40-mL cohort. DFA-02 was well tolerated and showed no evidence of local tissue reaction or impairment of wound healing. No serious AEs were deemed related to study drug. Systemic exposure to gentamicin and vancomycin remained well below levels considered to be at higher risk for oto- or nephrotoxicity. The maximal gentamicin and vancomycin levels observed were 2.36 and 0.684 μg/mL at 6 h, which were well below the prespecified stopping criteria of 12 and 20 μg/mL, respectively.

**Conclusions:**

In this small phase 2a study, the study drug was well tolerated and appeared to be free of serious adverse effects. Consistent with these findings, the PK values were consistent with gradual release of the antibiotics from the gel in the surgical site.

**Trial registration:**

ClinicalTrials.gov, NCT01496352

## Background

Surgical site infections (SSIs) are a common nosocomial infection in the USA and a significant cause of mortality and morbidity (Leaper & Ousey 2015; Leaper et al. 2015). Strategies to reduce SSI have focused on many factors, including, but not limited to, avoidance of shaving (Ko et al. 1992), appropriate selection, timing and dosing of prophylactic antibiotics (Bratzler et al. 2013), and avoidance of hypothermia (Kurz et al. 1996). However, despite widespread implementation of these strategies, SSIs remain common (Leaper & Ousey 2015), although there is some evidence that implementation of these bundles may be beginning to have a positive impact (Tanner et al. 2015).

An attractive potential strategy involves administration of a delayed release antibiotic into the surgical wound prior to the completion of surgery. A gentamicin-containing sponge has had mixed results; (Bennett-Guerrero et al. 2010; Bennett-Guerrero et al. 2010; Friberg et al. 2005; Rutten & Nijhuis PH. Prevention of wound infection in elective colorectal surgery by local application of a gentamicin-containing collagen sponge. Eur J Surg Suppl 1997) efficacy was not observed in some trials, perhaps due to the lack of a potent Gram-positive antimicrobial in the product. DFA-02 is a novel bioresorbable modified-release gel consisting of both gentamicin (16.8 mg/mL) and vancomycin (18.8 mg/mL). This sterile, viscous, clear gel contains sesame oil, soy lecithin, and dehydrated alcohol as excipients. The doses selected for this study were based on the antimicrobial activity, PK/pharmacodynamic (PD), and toxicologic results of preclinical studies conducted with prototype gentamicin/vancomycin gels and with DFA-02. From the PK findings in multiple animal studies, it was expected that the formulation of DFA-02, with 16.8 mg/mL gentamicin and 18.8 mg/mL vancomycin, would provide local tissue levels greater than four times the minimum inhibitory concentration (MIC) for susceptible Gram-positive and Gram-negative pathogens for up to 2 days after intraoperative application.

Therefore, the following phase 2a multicenter, randomized, double-blind, dose-ascending study was designed to test the safety, tolerability, and pharmacokinetics of DFA-02 in patients undergoing non-emergent colorectal surgery.

## Methods

This phase 2a study was sponsored/funded by Dr. Reddy’s Laboratories (Princeton, NJ) and coordinated by the Duke Clinical Research Institute (DCRI, Durham, NC), and was conducted at six US sites. It was registered at clinicaltrials.gov (NCT01496352).

Ethics, consent, permissions, and competing interests—the study was approved by the Duke Health System Institutional Review Board as well as the IRBs overseeing research at each of the sites as follows: The Ohio State University Medical Center (Western Institutional Review Board), Tampa General Hospital, and University of South Florida South Tampa Center (Western Institutional Review Board), Scott & White Healthcare (Scott & White Healthcare Institutional Review Board), Eliza Coffee Memorial Hospital (Western Institutional Review Board), Memorial Hermann Memorial City Medical Center (Western Institutional Review Board). All subjects provided written informed consent. Safety was reviewed on an ongoing basis by a colorectal surgeon (Walter Koltun, MD, Hershey Medical Center, Hershey, PA) not otherwise involved in the trial. This trial was funded by the sponsor (Dr. Reddy’s Laboratories); however, the DCRI controlled the performance of all data analysis and drafted the manuscript.

Adult patients undergoing colorectal surgery were enrolled in this trial.

Inclusion criteria were as follows: (1) males and non-pregnant females 18 years of age or older; (2) body mass index (BMI) 25 to 40; and (3) scheduled to undergo non-emergent colorectal surgery involving a laparotomy incision of 7 cm or greater (hand-assisted laparoscopic surgery was allowed). Eligible procedures included left, right, or transverse colectomy; segmental/sleeve left colon resection; total abdominal colectomy with ileorectal anastomosis; total abdominal colectomy with ileostomy; total abdominal proctocolectomy; low anterior resection; sigmoid resection; non-emergent Hartmann’s procedure; colotomy with polypectomy distal to hepatic flexure; colostomy takedown through laparotomy (not peristomal) incision; ileo-pouch anal anastomosis; and abdominal perineal resection of the rectum.

Exclusion criteria were as follows. (1) Known history of hypersensitivity to gentamicin or vancomycin, other aminoglycoside antibiotics, or the excipients of the study products (soy bean products or sesame oil). (2) Emergency surgery (urgent surgery was allowed if informed consent was obtained and the study procedures could be performed). (3) Significant concomitant surgical procedure (note: concomitant appendectomy, cholecystectomy, oophorectomy, and liver biopsy/wedge resection were allowed). (4) Prior laparotomy within 60 days of this planned procedure. (5) Planned second laparotomy or colorectal surgical procedure (e.g., colostomy or ileostomy takedown) within 30 days of this planned first procedure. (6) Expectation that a surgical drain would be placed. (7) Preoperative sepsis, severe sepsis, or septic shock. (8) Abdominal wall infection/SSI from previous laparotomy/laparoscopy or for any reason. (9) Active systemic infection or systemic (oral or intravenous) antibiotic therapy within 1 week before the date of surgery other than specified preoperative antimicrobial prophylaxis (note: single-dose antibiotic therapy for dental or other minor procedures was allowed as was the use of oral nonabsorbable antibiotics for preoperative bowel decontamination). (10) Requirement for gentamicin or vancomycin preoperative antimicrobial prophylaxis (note: systemic antibiotic therapy within 72 h after surgery with gentamicin or vancomycin was to be avoided, and any systemic antibiotic therapy during that time was to be discussed with the Coordinating Center Principal Investigator or Medical Monitor). (11) Requirement for concomitant use or use during the 30 days before day 1 of any prescription or over-the-counter drug that would interfere with the study or place the patient at undue risk. Concurrent systemic or topical use of other potentially neurotoxic, nephrotoxic, and/or ototoxic drugs, such as gentamicin, cisplatin, cephaloridine, kanamycin, amikacin, polymyxin B, colistin, paromomycin, streptomycin, tobramycin, vancomycin, ethacrynic acid, furosemide, and viomycin, was to be avoided. (12) Preoperative evaluation suggested an intra-abdominal process that might preclude full closure of the skin. (13) Ongoing treatment (e.g., chemotherapy, radiation) for non-colorectal cancer. (14) History of significant drug or alcohol abuse within the past year. (15) Serum creatinine >1.3 mg/dL. (16) Serum bilirubin >2.5 times upper limit of normal. (17) History of uncontrolled diabetes mellitus (controlled diabetic patients whose hemoglobin A1c was ≤9.0 % could be included). (18) Patients who were immunocompromised, including, but not limited to, systemic corticosteroid use or chemotherapy/radiation during the 30 days before surgery, organ transplantation, or human immunodeficiency virus infection (note: inhaled corticosteroids were not exclusionary, and single-dose use of corticosteroids to prevent postoperative nausea and vomiting was allowed). (19) Any clinically meaningful hearing loss (from medical history). (20) Clinically exclusionary results on clinical laboratory, electrocardiogram, or physical examination, including, but not limited to, positive hepatitis B or C or human immunodeficiency virus. (21) Pregnant or lactating or if of childbearing potential and not practicing a birth control method with a high degree of reliability. (22) Refusal to accept medically indicated blood products. (23) Participation within 30 days before the start of this study in any experimental drug or device study or currently participating in a study in which the administration of investigational drug or device within 60 days was anticipated. (24) Patients with anterior abdominal wall mesh that was not planned to be completely removed during the planned procedure. (25) Unable to participate in the study for any reason in the opinion of the PI. (26) Postsurgical life expectancy of less than 30 days, in the investigator’s or sponsor’s opinion. (27) Expected discharge from the hospital less than 3 days after surgery.

### Study drug

DFA-02 is a novel bioresorbable modified-release gel consisting of both gentamicin (16.8 mg/mL) and vancomycin (18.8 mg/mL). This sterile, viscous, clear gel contains sesame oil, soy lecithin, and dehydrated alcohol as excipients. The placebo gel was exactly the same as DFA-02 except for the active ingredients. The products were manufactured by NextPharma, Inc. (San Diego, CA). DFA-02 and matching placebo were supplied as 10 mL in 20-mL glass vials. The product was stored at −25 to −10 °C. It was thawed at room temperature until clear and used within 7 days of thawing. It was not refrozen and reused.

### Randomization and study drug administration

Patients were randomized using a computer generated randomization scheme prepared by the trial’s statistician at the DCRI. DFA DFA-02 10, 20, or 30 mL or matching placebo was administered once at the conclusion of surgery in the operating room. The gel from one or two vials (10 or 20 mL) was drawn into a 30-mL syringe (provided by the sponsor) from the vial with a 14-gauge needle (also provided by the sponsor) or, alternatively, the gel from the vial could be decanted into a sterile container/cup and drawn into the syringe without a needle. For the 10- and 20-mL cohorts, a single 30-mL syringe was used, and for the 30-mL cohort, two 30-mL syringes were used. After closure of the fascia, the gel was administered from the syringe, evenly covering the subcutaneous tissue of the index incision (the longest incision if more than one incision was made). The subcutaneous tissue was then approximated and the incision closed with either sutures or staples. Any extruded gel after closure was collected in a syringe, the volume recorded by the surgeon, and then discarded. Only the longest (index) laparotomy incision was treated with study drug. If a patient required reopening of the surgical incision for any reason, no additional study drug was applied.

### Prohibited and concomitant medications/procedures

Consistent with the Surgical Care Improve Project (SCIP) guidelines, all patients received standard preoperative antimicrobial prophylaxis antibiotics within 60 min of initial skin incision. Antibiotic prophylaxis was not continued for more than 24 h. Antibiotic prophylaxis could be redosed during surgery for long procedures based on SCIP guidelines. The use of oral nonabsorbable preoperative antibiotic treatment was at the discretion of the surgeon. Adherence to other SCIP guidelines, e.g., maintenance of normothermia, method of hair removal, was recorded. Prohibited interventions included administration of topical antimicrobials (e.g., Betadine), systemic gentamicin or vancomycin preoperatively or within 72 h of surgery, and prophylactic use of negative pressure dressings (e.g., WoundVac, StripVac).

### Outcomes/recorded variables

The main objectives of the study were to (1) evaluate the safety and tolerability of ascending doses of DFA-02 in patients undergoing colorectal surgery and (2) evaluate the systemic PK of gentamicin and vancomycin after single doses of 10 to 40 mL of DFA-02 in patients undergoing colorectal surgery.

Safety and tolerability were assessed by (1) physical examination; (2) surgical incision examination on days 2, 3, and 5 (24, 48, and 96 h postoperatively) and day 14; (3) vital signs: screening (baseline), day of surgery (pre- and postoperatively), days 2, 3, 5, and 14; (4) clinical laboratory tests (hematology, clinical chemistry, urinalysis): screening, days 5 and 14; (5) ECG: screening (baseline), day 5; (6) adverse event recording on days 1, 2, 3, 5, 14, and 30; (7) ASEPSIS score on day 5 (96 h) postoperatively; and (8) wound questionnaire on day 30.

Additional recorded variables included (1) evaluation of postoperative renal function as manifested by serum creatinine levels on days 5 and 14 compared with baseline and (2) evaluation of the incidence of antibiotic resistance based on baseline and postoperative rectal/stool and nasal culture and sensitivities for vancomycin-resistant enterococcus (VRE) and MRSA, respectively, and antibiotic sensitivity of any culture positive SSIs. Molecular typing was also utilized to identify MRSA and VRE in lieu of culture and sensitivity.

Systemic PK of gentamicin and vancomycin were determined from serial blood draws obtained at 1, 6, 24, 48, and 96 h after study drug administration. These assays were performed at MicroConstants, Inc (San Diego, CA).

### Statistical methods

The sample size and study design were typical for phase 2 safety, tolerability, and PK studies, and the study was not statistically powered. This study planned to enroll 40 patients (ten per cohort) undergoing colorectal surgery with a planned incision of 7 cm or greater. Four dose cohorts were planned: 10, 20, 30, and 40 mL of DFA-02 administered as a single dose into the surgical incision at closure. All study data were summarized using descriptive statistics at each assessment time for each treatment group based on actual values and change from baseline/screening values. Continuous variables were summarized using *n*, mean, standard deviation (SD), and minimum, median, and maximum values. Categorical variables were summarized using the number and percentage of patients in each category. Gentamicin and vancomycin plasma levels were obtained 1 and 6 h after study drug application and on days 2, 3, and 5 (24, 48 and 96 h after application). *C*_max_, AUC_0–*t*_, AUC_0–∞_, *T*_max_, *t*_1/2_, CL/F, and V/F were calculated from the plasma concentrations using standard methods.

## Results

Study enrollment was discontinued after the first three dose cohorts (10, 20, and 30 mL) as even very large incisions could not accommodate more than 20 mL of gel, leaving no scientific justification for the 40 mL cohort. Each cohort contained ten subjects (two patients receiving placebo and eight patients receiving study drug). It was anticipated that not all of the study drug would be retained in the wound, e.g., smaller incisions, so we recorded the net volume of study drug retained. Therefore, results (Table 1) are presented for subjects with ≤10 mL vs >10 mL retained study drug.

**Table 1:** Patient and procedure characteristics and study drug administration

Characteristic	Placebo	DFA-02 ≤10 mL	DFA-02 >10 mL
	(*n* = 6)	(*n* = 16)	(*n* = 8)
Age, years	56.5 (45.1,60.6)	70.9 (63.2, 76.3)	46.9 (41.4, 61.2)
Gender, % male	2 (33.3 %)	6 (37.5 %)	4 (50 %)
Race, % white	5 (83.3 %)	14 (87.5 %)	7 (87.5 %)
Weight, kg	74.7 (72.3,76.4)	90.9 (72.3,97.1)	81.8 (73.3, 97.5)
Height, cm	164.3 (157.5, 175.2)	167.6 (158.7, 175.3)	171.4 (165.1, 180.4)
Body mass index (kg/m^2^)	28.2 (27.0, 29.9)	29.1 (27.8, 33.9)	28.2 (25.7, 30.6)
Preoperative serum creatinine (mg/dL)	0.7 (0.7, 0.9)	0.9 (0.8, 1.3)	0.9 (0.8, 1.0)
Duration of surgical procedure (h)	2.8 (1.4, 4.6)	2.0 (1.1, 2.3)	3.0 (2.4, 5.6)
Length of incision (cm)	11.3 (8.0, 26.0)	8.2 (7.8, 10.0)	8.5 (7.8, 10.5)
Depth of incision at midpoint (cm)	2.2 (1.5, 3.0)	2.5 (1.5, 3.3)	2.5 (2.0, 3.0)
Wound area (cm^2^)	45.7 (11.1, 79.7)	22.3 (12.1, 30.0)	22.8 (18.0, 32.3)
Study drug volume inserted into wound (mL)	19.0 (10.0, 20.0)	10.0 (10.0, 20.0)	25.0 (20.0, 30.0)
Overflow study drug collected (mL)	4.5 (3.0, 7.0)	5.0 (1.5, 12.5)	12.0 (6.0, 15.0)
Volume of study drug retained in wound (mL)	9.5 (5.0, 17.0)	5.0 (4.5, 9.8)	15.0 (12.5, 15.5)
Volume of study drug per wound area (mL/cm^2^)	0.2 (0.2, 0.5)	0.3 (0.2, 0.4)	0.6 (0.5, 0.7)

As appropriate data shown as percentage or median (25th, 75th percentiles)

Table 1 shows preoperative and intraoperative variables. The study dose groups showed some differences in distributions of baseline and intraoperative characteristics consistent with the relatively small sample size inherent to the phase 2 design.

DFA-02 was well tolerated and showed no evidence of local tissue reaction or impairment of wound healing. The majority of treatment-emergent adverse events (TEAEs) were those commonly seen with colorectal surgery. No serious AEs were considered to be related to study drug. Serum creatinine measured on postoperative days 4 (or discharge) and 14 were not significantly increased compared with baseline values. No patients showed MRSA in preoperative nasal cultures or VRE in preoperative rectal cultures. One patient in the DFA-02 group showed nasal MRSA and one patient in the placebo group showed rectal VRE at discharge.

**Fig. 1:**
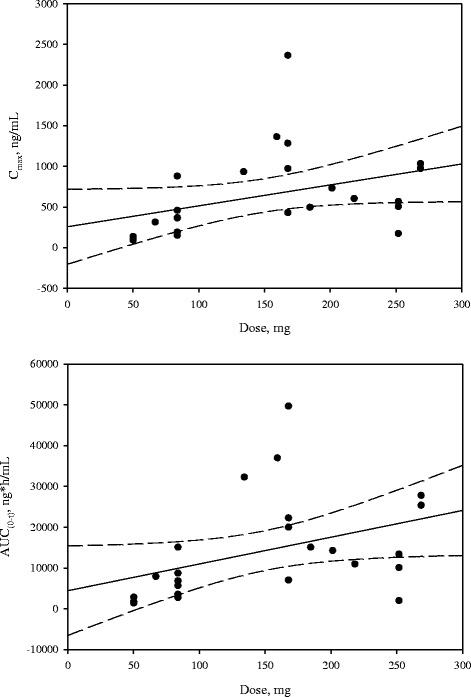
**a** and **b** show the *C*_max_ and AUC_0–t_ of gentamicin following a single administration of DFA-02 (actual dose)

**Fig. 2:**
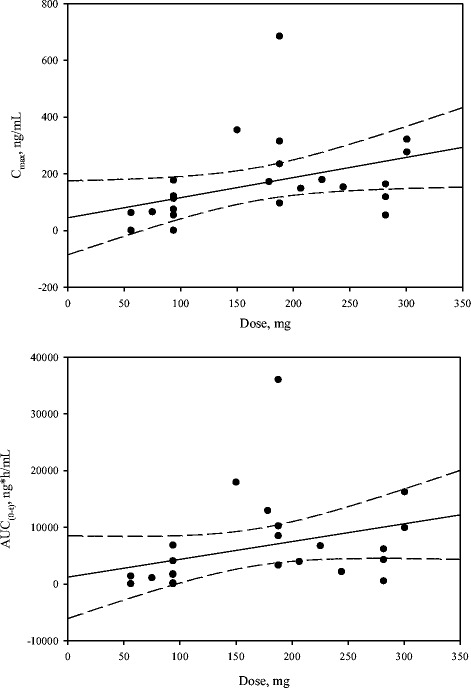
**a** and **b** show the *C*_max_ and AUC_0–t_ of vancomycin following a single administration of DFA-02 (actual dose)

Systemic exposure to gentamicin and vancomycin remained well below levels considered to be at higher risk for oto- or nephrotoxicity. The maximal gentamicin level for any patient was 2.36 μg/mL at 6 h, which was well below the prespecified stopping criterion of 12 μg/mL at 6 h. The maximal vancomycin level for any patient was 0.684 μg/mL at 6 h, well below the prespecified stopping criterion of 20 μg/mL. Pharmacokinetic data are shown in Table 2 and Figs. 1 and 2. The PK analysis showed that following single administration of DFA-02 gel at 10, 20, or 30 mL (nominal volume) to surgical wounds, gentamicin was absorbed into the systemic circulation with median *T*_max_ values of 6.00, 6.09, 6.15, and 6.01 h for cohorts 1, 2, 3, and all cohorts combined, respectively. The *T*_max_ values appeared to be dose-independent. The mean *C*_max_ values were 0.693, 0.626, 0.608, and 0.642 μg/mL for cohorts 1, 2, 3, and all cohorts combined, respectively. Corresponding mean AUC_0–t_ values were 15.30, 15.20, 12.10, and 14.20 μg∙h/mL for cohorts 1, 2, 3, and all cohorts combined, respectively. Vancomycin was absorbed into the systemic circulation with median *T*_max_ values of 6.57, 23.9, 24.1, and 22.2 h for cohorts 1, 2, 3, and all cohorts combined, respectively. The median *T*_max_ in cohorts 2 and 3 was longer than that of cohort 1. The mean *C*_max_ values were 0.157, 0.168, 0.166, and 0.164 μg/mL for cohorts 1, 2, 3, and all cohorts combined, respectively. Corresponding mean AUC_0–t_ values were 6.41, 6.28, 6.71, and 6.47 μg h/mL in cohorts 1, 2, 3, and all cohorts combined, respectively.

**Table 2:** Summary of gentamicin and vancomycin pharmacokinetic parameters in all cohorts

Characteristic	Actual dose^a^	*T*_max_	*C*_max_	AUC_(0–t)_	*t*_1/2_
	(mg)	(h)	(mcg/mL)	(mcg h/mL)	(h)
Gentamicin					
*n*	24	24	24	24	18
Mean (SD)	149 (75.9)	6.01 (3.48)	0.642 (0.524)	14.2 (12.6)	17.7 (4.98)
Vancomycin					
*n*=	24	21	24	24	0
Mean (SD)	167 (84.9)	22.2 (18.7)	0.164 (0.150)	6.47 (8.12)	NC

*AUC* area under the curve, *C*_max_, maximum concentration, *h* hour, *mcg* microgram, *mg* milligram, *mL* milliliter, *N* number, *NC* not calculated, *SD* standard deviation, *t*_½_ half-life

^a^Mean amount of drug retained for all cohorts 8.9 ± 4.52 mL

The study was not designed or powered to rigorously assess SSIs; however, no significant differences were observed in this endpoint (placebo 1/6 = 16.7 %, DFA-02 ≤10 mL retained dose 3/16 = 18.8 %, DFA-02 >10 mL retained dose 2/8 = 25 % SSI rate).

## Discussion

This phase 2 dose-ascending tolerability, safety, and pharmacokinetic study met its objectives. The inclusion of a placebo group in each dosing cohort enabled us to maintain a double-blind study, avoiding possible bias with regard to assessments of safety and tolerability. Consistent with preclinical animal studies conducted by the sponsor, DFA-02 was well tolerated and showed no evidence of local tissue reaction or impairment of wound healing. Safety was assessed by collection of adverse events and by recording safety events of interest, e.g., related to renal function and serum levels of the antibiotics. No serious adverse events were deemed related to study drug.

Postoperative renal function, assessed by mean serum creatinine levels, showed no adverse effect for DFA-02 compared with placebo gel. A single patient had a creatinine elevation 2 days after surgery that returned to normal within 3 days. The maximum recommended daily intravenous dose for gentamicin in patients with normal renal function is 1.7 mg/kg every 8 h or 306 mg/day for a 60-kg patient with a goal of avoiding peak systemic levels greater than 12 μg/mL and trough levels greater than 2 μg/mL (Gentamicin Dosage - Drugs.com. Available at: http://www.drugs.com/dosage/gentamicin.html#Usual_Adult_Dose_for_Surgical_Prophylaxis. Accessed July 2 2013). The usual recommended IV dose for vancomycin is 2 g/day with a therapeutic goal of 15 to 20 μg/L (Vancomycin Dosage - Drugs.com. Available at: http://www.drugs.com/dosage/vancomycin.html. Accessed July 2 2013). In both rabbit and pig models of wound healing, systemic concentrations of gentamicin have been below the systemic peak level, 12 μg/mL, above which nephro- and ototoxicity are more likely to occur, and vancomycin concentrations have been below 20 μg/mL. The highest maximum concentration (*C*_max_)/DFA-02 dose correlations observed in preclinical in vivo studies were 0.69 μg/mL for gentamicin and 0.33 μg/mL for vancomycin, observed in a pig model of wound healing (Study MPI 1115-019, Dr. Reddy’s Laboratories). Extrapolating from the *C*_max_/dose correlations in pigs, the expected human *C*_max_ for a 60-kg person receiving 40 mL of DFA-02 was estimated to be 7.8 μg/mL for gentamicin and 4.2 μg/mL for vancomycin, both within the clinically accepted range. In our study, the maximal levels of gentamicin and vancomycin for any patient were 2.36 and 0.684 μg/mL at 6 h, respectively, well below the prespecified stopping criteria for the study. In fact, the maximum dose actually tested was 30 mL since as even very large incisions could not accommodate more than 20 mL of gel, leaving no scientific justification for advancing dose escalation to the 40 mL cohort. The systemic PK suggested slow release of the antibiotics from the gel in the incision site.

Our study has several limitations. As is typical for most “first in patient” studies, in order to optimize safety of study subjects, there was an extensive list of exclusion criteria, which can limit the generalizability of these results to all surgical patients. In particular, while preclinical models showed no adverse effects of the study drug on renal function, to be conservative, we excluded patients with serum creatinine >1.3 mg/dL preoperatively. While no adverse effect was shown on renal function, we cannot rule out different results in patients with preexisting renal dysfunction. As expected, and consistent with the small sample size, we observed imbalances between study arms in several patient characteristics. It is important to note, however, that this should not affect many of the study’s findings given the nature of the pharmacokinetic and other analyses. Finally, our study was not powered to draw any conclusions as to the efficacy of DFA-02 in preventing SSIs in colorectal surgery. A larger multicenter study is designed to evaluate the efficacy of DFA-02 with respect to prevention of SSI.

## Conclusions

In this small phase 2a study, the study drug was well tolerated and appeared to be free of serious adverse effects. Consistent with these findings, the PK values were consistent with gradual release of the antibiotics from the gel in the surgical site.

## Abbreviations

AUC, area under curve; BMI, body mass index; MIC, minimum inhibitory concentration; MRSA, methicillin resistant *Staphylococcus aureus*; PD, pharmacodynamics; PK, pharmacokinetics; SCIP, Surgical Care Improvement Project; SD, standard deviation; SSI, surgical site infection; VRE, vancomycin-resistant enterococcus
